# 2017/18 and 2018/19 seasonal influenza vaccine safety surveillance, Canadian National Vaccine Safety (CANVAS) Network

**DOI:** 10.2807/1560-7917.ES.2020.25.22.1900470

**Published:** 2020-06-04

**Authors:** Julie A Bettinger, Gaston De Serres, Louis Valiquette, Otto G Vanderkooi, James D Kellner, Brenda L Coleman, Karina A Top, Jennifer E Isenor, Anne E McCarthy, on behalf of the Canadian Immunization Research Network

**Affiliations:** 1 Vaccine Evaluation Center, BC Children’s Hospital, University of British Columbia, Vancouver, Canada; 2 CHU de Québec-Université Laval, Québec City, Canada; 3 Centre Intégré Universitaire de Santé et de Services Sociaux de l’Estrie- Centre Hospitalier Universitaire de Sherbrooke, Sherbrooke, Canada; 4 Department of Pediatrics and Alberta Children’s Hospital Research Institute, University of Calgary, Calgary, Canada; 5 Sinai Health System and University of Toronto, Toronto, Canada; 6 Canadian Center for Vaccinology, IWK Health Centre and Department of Pediatrics, Dalhousie University, Halifax, Canada; 7 College of Pharmacy and Canadian Center for Vaccinology, Dalhousie University, Halifax, Canada; 8 Department of Medicine, Ottawa Hospital, Ottawa, Canada; 9 The Canadian Immunization Research Network is acknowledged at the end of this article

**Keywords:** AEFI, influenza vaccine, safety signal, online monitoring

## Abstract

**Background:**

The Canadian National Vaccine Safety (CANVAS) network monitors the safety of seasonal influenza vaccines in Canada.

**Aim:**

To provide enhanced surveillance for seasonal influenza and pandemic influenza vaccines.

**Methods:**

In 2017/18 and 2018/19 influenza seasons, adults (≥ 15 years of age) and parents of children vaccinated with the seasonal influenza vaccine participated in an observational study using web-based active surveillance. Participants completed an online survey for health events occurring in the first 7 days after vaccination. Participants who received the influenza vaccine in the previous season, but had not yet been vaccinated for the current season, were unvaccinated controls.

**Results:**

In 2017/18, 43,751 participants and in 2018/19, 47,798 completed the online safety survey. In total, 957 of 30,173 participants vaccinated in 2017/18 (3.2%; 95% confidence interval (CI): 3.0–3.4) and 857 of 25,799 participants vaccinated in 2018/19 (3.3%; 95% CI: 3.1–3.5) reported a health problem of sufficient intensity to prevent their normal daily activities and/or cause them to seek medical care (including hospitalisation). This compared to 323 of 13,578 (2.4%; 95% CI: 2.1–2.6) and 544 of 21,999 (2.5%; 95% CI: 2.3–2.7) controls in each respective season. The event rate in vaccinated adults and children was higher than the background rate and was associated with specific influenza vaccines. The higher rate of events was associated with systemic symptoms and migraines/headaches.

**Conclusion:**

In 2017/18 and 2018/19, higher rates of events were reported following seasonal influenza vaccination than in the pre-vaccination period. This signal was associated with several seasonal influenza vaccine products.

## Introduction

As one of the most successful public health interventions of the last century, modern vaccines are safe and effective [1]. Unlike other medications, vaccines are given to prevent diseases rather than to treat them, often to healthy people. Therefore, low tolerance exists for adverse events following immunisation (AEFI) requiring effective surveillance and monitoring of post-market effects. Implementation of effective pharmacovigilance supports public and provider confidence in vaccines and vaccination programmes.

In Canada, current vaccine pharmacovigilance involves both passive and active surveillance (monitoring) systems [2] that are designed to detect, at minimal cost, very rare events in the large population of recipients of a broad range of vaccines. Passive surveillance suffers from under-reporting and reporting bias (based on age and severity) [3]. Active surveillance occurs only in children (< 17 years of age) for specific conditions, such as seizures [4,5]. Both types of monitoring can be slow to recognise safety signals and neither permits the calculation of population-based incidence rates of AEFI. Finally, neither system is adequately designed to provide enhanced reporting for influenza vaccines, which contain new antigens almost every year, with millions of doses administered over a short time period.

The Internet and mobile technology offer powerful tools for large-scale, direct reporting of health events [6-11]. They allow for rapid identification of AEFI with minimal human resource needs and overcome some of the shortcomings of passive surveillance (i.e. inability to calculate population-based rates). In spite of the potential, online vaccine safety surveillance requires careful calibration to determine the minimal amount of information required to detect and interpret safety signals while still maintaining participant engagement. Further, while those who experience an AEFI may be motivated to respond, the response of those who do not develop AEFI remains critical.

Since 2009 electronic surveillance of the occurrence of adverse events following influenza vaccination has been conducted across Canada [9,10]. The Canadian National Vaccine Safety (CANVAS) network is designed to quickly gather and analyse safety data on thousands of vaccinated individuals (adults and children) to provide influenza vaccine safety information to public health authorities before the core weeks of the annual influenza vaccination campaign, when influenza vaccination reaches its seasonal peak. In 2012, more than 8,000 adults participated in this web-based active surveillance and that year it was expanded to include children [12]. By 2013, the surveillance included over 35,000 vaccinated and unvaccinated adults and children in Canada. This report summarises the results from participants surveyed in 2017/18 and 2018/19 influenza seasons. The influenza vaccine strains used in these seasons differed. In 2017/18 recommended strains were an A/Michigan/45/2015 (H1N1)pdm09-like virus; an A/Hong Kong/4801/2014 (H3N2)-like virus; a B/Brisbane/60/2008-like virus (B/Victoria/2/87 lineage) and, for the quadrivalent vaccines, a B/Phuket/3073/2013-like virus (B/Yamagata/16/88 lineage). In 2018/19 recommended strains were an A/Michigan/45/2015 (H1N1)pdm09-like virus; an A/Singapore/INFIMH-16–0019/2016 (H3N2)-like virus; a B/Colorado/06/2017/18-like virus (B/Victoria/2/87 lineage); and, for the quadrivalent vaccines, a B/Phuket/3073/2013-like virus.

## Methods

### Recruitment of vaccinees and controls

The 2017/18 and 2018/19 surveillance recruited adults (≥ 15 years of age) and children from the Canadian provinces of Alberta (Calgary metropolitan area), British Columbia (Vancouver metropolitan area), Nova Scotia (Halifax), Ontario (Ottawa and Toronto metropolitan areas) and Quebec (Quebec City and Sherbrooke). The surveillance has been active since 2009 and for 2017/18 and 2018/19 it included a convenience sample of hospital, pharmacy, public health, university, and workplace clinics. Criteria for site inclusion included early initiation of influenza vaccination clinics (i.e. immediately after the release of seasonal influenza vaccines in each jurisdiction) and vaccination of several hundred individuals per day with the first 2 weeks following vaccine release. Vaccinees were recruited at the time of vaccination and enrolment consisted of obtaining contact information for follow up. Eligibility criteria included receipt of influenza vaccine at an ambulatory clinic, ability to understand the study request in English or French and access to email and telephone. Vaccinated participants were eligible to participate annually, but were required to re-enrol each year at the time of vaccination and their surveys were not linked from year to year. The unvaccinated control participants were the previous year’s vaccinated participants who agreed to participate as unvaccinated controls the following autumn and were re-contacted before the start of the seasonal influenza vaccination programme with their prior consent.

### Survey procedures

Both groups received a survey link to an online questionnaire that measured health events occurring in the previous 7 days. Vaccinees received the survey link 8 days after vaccination. Controls received an email 14–21 days before the start of influenza vaccination programmes asking them to note health events in the following 7 days and then they received the control survey link 8 days after the monitoring email. Non-responders in both groups were sent a reminder survey link 3 days after the original link.

Data were collected on a rolling basis because each provincial influenza vaccination programme starts at a different time. In 2017/18 and 2018/19 the earliest vaccination programme started on 3 October while the latest programme started on 1 November. Each site recruited vaccinated participants for 2 weeks.

### Description of questionnaires and outcomes

The online questionnaires collected information on demographics (i.e. age, gender), past influenza vaccination history and occurrence of health events of interest. Age was solicited in the following age groups for children: 6–23 months; 2–4 years; 5–9 years; and 10–14 years. In adults, it was 15–19 years and in 10-year age bands for adults ≥ 20 years of age. Parents completed reports for their children 6 months to 14 years of age. The main outcomes were health events that prevented daily activities or resulted in work or school absenteeism, and/or required a medical consultation, including hospitalisation. The online survey collected these health events occurring (or worsening for existing conditions) in the 7 days after the email to controls or 7 days after vaccination for vaccinees.

The survey captured events reportable to public health and/or associated with previous influenza vaccines. Events were documented by broad categories: allergic-like events (hives, rash, swelling including facial swelling, swelling of the eyes (without bilateral red eyes), throat or tongue swelling with difficulty breathing or swallowing, wheezing); anaphylaxis; gastrointestinal symptoms (diarrhoea, nausea, stomach pain or vomiting); headache/migraine and seizures; change in eating (child survey only); local injection site reactions (vaccinated group only); systemic symptoms (fever ≥ 38.0^◦^C, fatigue, myalgia); respiratory symptoms suggestive of bronchitis, common cold, influenza, pharyngitis, pneumonia, sinusitis, tonsillitis. A text field was provided for other health events. Symptoms of oculorespiratory syndrome and numbness (anaesthesia/paresthesia) with onset in the first 24 hours of the study period were also solicited. Oculorespiratory syndrome was defined according to the following definition: onset within 24 hours of vaccination (for vaccinees) or within the past 24 hours (for controls) of bilateral red eyes and one of the following: eye-itchiness, eye-swelling, face-swelling, hard-to-swallow, rash with/without itchiness, respiratory symptoms (breathing-difficulty and/or wheezing, chest-tightness, cough, hoarseness), sore-throat, throat-swelling, tongue-swelling. Participants were not directly asked if they had oculorespiratory syndrome.

Medically-attended events triggered a telephone call within 72 hours of the online report, when possible, by a nurse or research assistant trained in eliciting AEFI information. Events reported in the text field of the ‘other event’ category were reviewed daily with unusual events or neurologic events followed up by phone. Events that met the criteria for public health reporting were reported to the local public health authorities for appropriate follow up.

### Statistical analysis

The frequency, proportion and 95% confidence intervals (CI) were calculated for events in the control and vaccinated groups. Age-standardised incidence rate ratios and 95% CIs were calculated for specific events and specific influenza vaccine products when the event occurred in both the vaccinated and control groups. The age distribution of the control group of each season was used to define the standard population. Incidence rate ratios and the 95% CIs were used to determine whether differences in event rates between control and vaccinated responders were significantly different at a 0.05 level with no adjustment for multiple comparisons. Self-report and proxy report (i.e. parents reporting events in their children 6 months to 14 years of age) were given equal weight in the analysis. Analyses were done using SAS v9.4.

### Ethical statement

All participants provided informed consent electronically. The Research Ethics Board at each participating site approved the study (REB15–0570_REN4 (Calgary); 2011–275, 10–156 (Sherbrooke); 09–0258E, 13–249, 768–1810-INF-042, 2018–08 and 17–0031 (Toronto); 1002988 and 1020286 (Halifax); MP-20–2014–1773, B13–08–1773 (Quebec City); 2010715–01H (Ottawa); H10–02274 (Vancouver)). The planning, conduct and reporting of the study was in line with the Declaration of Helsinki, as revised in 2013.

## Results

Among the 82,770 enrolled individuals vaccinated between 10 October and 27 November 2017 and between 3 October and 29 November 2018, 55,972 (67.6%) completed the online survey. Among the 72,672 previous participants contacted to be controls, 35,577 (49.0%) responded to the survey. The majority of non-responders in both groups did not click the survey link (Supplementary Figure 1).

### Participant characteristics

The majority of participants who completed survey were adults 15–64 years of age (56.4%; 51,676/91,549) followed by adults 65 years of age and older (29.8%; 27,242/91,549) (Table 1). The majority of participants were female (61.2%; 55,996/91,549; Table 1). Four per cent of adult vaccinees (1,760/47,908) and 6.9% of paediatric vaccinees (559/8,064) had not received an influenza vaccine in the prior 2 years. In adults, standard-dose trivalent inactivated vaccine (Fluviral, GSK) was the product most frequently administered (38.6% (9,944/25,786) in 2017/18 and 52.1% (11,535/22,122) in 2018/19) whereas, in children, quadrivalent inactivated vaccine (Fluzone, Sanofi Pasteur) was the most frequent (65.1% (2,855/4,387) in 2017/18 and 53.1% (1,954/3,677) in 2018/19).

**Table 1:** Characteristics of surveillance population Canada, 2017/18 and 2018/19 (n = 91,549^a^)

Characteristics of participants	2017/18						2018/19						Total 2017 and 2018					
	Controls			Vaccinees			Controls			Vaccinees			Controls			Vaccinees		
	N	%^b^	95% CI	N	%^b^	95% CI	N	%^b^	95% CI	N	%^b^	95% CI	N	%^b^	95% CI	N	%^b^	95% CI
**Participants enrolled**																		
Total	28,938	NA	NA	44,373	NA	NA	43,734	NA	NA	38,397	NA	NA	72,672	NA	NA	82,770	NA	NA
**Participants who completed online safety survey**																		
Total	13,578	46.9^c^	NA	30,173	68.0^c^	NA	21,999	50.3^c^	NA	25,799	67.2^c^	NA	35,577	49.0^c^	NA	55,972	67.6^c^	NA
Female	8,345	61.5	NA	18,390	60.9	NA	13,418	61.0	NA	15,843	61.4	NA	21,763	61.2	NA	34,233	61.2	NA
Number of vaccines in past 2 years	ND	NA	NA	1,352	4.5	NA	ND	NA	NA	967	3.7	NA	ND	NA	NA	2,319	4.1	NA
**Age group completing survey**																		
6 months–4 years	483	3.6	NA	1,871	6.2	NA	1,076	4.9	NA	1,771	6.8	NA	1,559	4.4	NA	3,642	6.5	NA
5–14 years	970	7.1	NA	2,516	8.3	NA	2,038	9.2	NA	1,906	7.4	NA	3,008	8.5	NA	4,422	7.9	NA
15–64 years	8,004	58.9	NA	17,369	57.6	NA	11,676	53.1	NA	14,627	56.7	NA	19,680	55.3	NA	31,996	57.2	NA
≥ 65 years	4,121	30.4	NA	8,417	27.9	NA	7,209	32.8	NA	7,495	29.1	NA	11,330	31.8	NA	15,912	28.4	NA
**Reported a health event^d^**																		
Total	323	2.4	2.1–2.6	957	3.2	3.0–3.4	544	2.5	2.3–2.7	857	3.3	3.1–3.5	867	2.4	2.3–2.6	1,814	3.2	3.1–3.4
Prevented daily activities or missed school/work	228	1.7	1.5–1.9	686	2.3	2.1–2.4	398	1.8	1.6–2.0	614	2.4	2.2–2.6	626	1.8	1.6–1.9	1,300	2.3	2.2–2.5
Medical consultation no absenteeism	34	0.3	0.2–0.3	56	0.2	0.1–0.2	50	0.2	0.2–0.3	60	0.2	0.2–0.3	84	0.2	0.2–0.3	116	0.2	0.2–0.2
Prevented daily activities and missed work/school and medical consultation	61	0.4	0.3–0.6	215	0.7	0.6–0.8	96	0.4	0.4–0.5	183	0.7	0.6–0.8	157	0.4	0.4–0.5	398	0.7	0.6–0.8

CI: confidence interval; NA: not applicable; ND: not determined (question was not asked of this group).

^a^ While the table covers the 155,442 individuals who were enrolled in the survey, the total number of individuals who completed the online safety survey is 91,459.

^b^ Unless otherwise stated (footnote c), percentages are calculated using as denominator the total number of survey participants who completed the online safety survey.

^c^ Percentage among the number of individuals enrolled in the survey for each category (i.e. control or vaccinee in each season).

^d^ A health event was defined by the respondent as of sufficient intensity to cause a medical consultation or work/school/day care absenteeism/prevent daily activities, or both.

### Reported health event rates

Between 2017/18 and 2018/19 the overall rate of health events meeting the main outcome criteria did not differ significantly among vaccinated participants at 3.2% (95% CI: 3.0–3.4) and 3.3% (95% CI: 3.1–3.5) in 2017/18 and 2018/19, respectively (Table 1). However, the rate was significantly different between vaccinated and control participants each season, with the rate in controls being 2.4% (95% CI: 2.1–2.6) in 2017/18 and 2.5% (95% CI: 2.3–2.7) in 2018/19 (Table 1). Each year, health event rates were significantly higher in adult vaccinees than in adult controls, and in children compared to adults (Table 2). In both years, the health event rates were not significantly different between vaccinated children and control children (Table 2). Additionally, the percentage of children who missed school or daily activities and sought medical care was slightly higher among vaccinees in 2018/19 when compared with controls (Table 2), while the percentage who only sought medical care was the same among vaccinated and control children for both years (Table 2). Overall, the percentage of children seeking medical care was 2.1% in both years (1.8%+0.3%; Table 2).

**Table 2:** Health event^a^ rate in adults^b^ and children^b^, Canada, 2017/18 and 2018/19 (n = 91,549)

Participant characteristic, type of health event	2017/18				2018/19			
	Number reporting a health event	Total	%	95% CI	Number reporting a health event	Total	%	95% CI
Adults^b^								
Control, health events	235	12,125	1.9	1.7–2.2	381	18,885	2.0	1.8–2.2
Vaccinee, health events	656	25,786	2.5	2.4–2.7	614	22,122	2.8	2.6–3.0
Children^b^								
Control, health events	88	1,453	6.1	4.9–7.4	163	3,114	5.2	4.5–6.1
Vaccinee, health events	301	4,387	6.9	6.1–7.6	243	3,677	6.6	5.8–7.5
o Control, medical consultation and activities prevented/school absenteeism	19	1,453	1.3	0.8–2.0	31	3,114	1.0	0.7–1.4
o Vaccinee, medical consultation and activities prevented/school absenteeism	80	4,387	1.8	1.4–2.3	67	3,677	1.8	1.4–2.3
o Control, medical consultation	4	1,453	0.3	0.1–0.7	10	3,114	0.3	0.2–0.6
o Vaccinee, medical consultation	15	4,387	0.3	0.2–0.6	12	3,677	0.3	0.2–0.6

CI: confidence interval.

^a^ A health event was defined by the respondent as of sufficient intensity to cause a medical consultation or work/school/day care absenteeism/prevent daily activities, or both.

^b ^A person is considered as an adult if aged ≥ 15 years; children are aged 6 months–14 years.

### Health event rates by vaccine product

The higher rate of health events in vaccinees occurred with six specific products (Table 3): standard-dose trivalent inactivated vaccine (Agriflu, Seqirus; Fluviral, GSK; and Influvac, Abbott) and quadrivalent inactivated vaccine (Fluzone, Sanofi-Pasteur; and Flulaval, GSK) and high-dose trivalent inactivated vaccine (Fluzone High-Dose, Sanofi-Pasteur). Some variation existed when examined by specific age groups (Table 3).

**Table 3:** Incidence and incidence rate ratios of health events^a^ in vaccinees compared with controls, Canada 2017/18 and 2018/19 (n = 91,549)

Type and number of participants with vaccine administered where applicable	Incidence or IRR of health events with 95% CI per age group								Total	
	6 months–4 years		5–14 years		15–64 years		≥ 65 years			
2017/18	Incidence	95% CI	Incidence	95% CI	Incidence	95% CI	Incidence	95% CI	Standardised incidence	95% CI
Controls n = 13,578	8.70	6.34–11.57	4.74	3.49–6.28	2.44	2.11–2.80	0.97	0.69–1.32	2.44	2.28–2.61
Vaccinees n = 30,173	8.82	7.57–10.20	5.41	4.55–6.36	3.17	2.92–3.44	1.25	1.02–1.51	NA	NA
2017/18	IRR	95% CI	IRR	95% CI	IRR	95% CI	IRR	95% CI	Standardised IRR	95% CI
Agriflu trivalent (Seqirus) n = 4,992	**3.07**	**1.26–7.45**	1.05	0.15–7.27	0.77	0.57–1.04	1.06	0.63–1.79	**1.21**	**1.11–1.32**
FluLaval quadrivalent (GSK) n = 462	0.83	0.51–1.36	1.41	0.58–3.43	NA	NA	NA	NA	0.36	0.32–0.41
FluMist quadrivalent (AstraZeneca) n = 964	0.77	0.47–1.28	0.67	0.40–1.14	NA	NA	NA	NA	0.72	0.61–0.86
Fluviral trivalent (GSK) n = 10,091	0.98	0.37–2.61	0.42	0.10–1.71	**1.28**	**1.05–1.56**	1.28	0.84–1.95	**1.09**	**1.00–1.20**
Fluzone quadrivalent (Sanofi Pasteur) n = 8,972	1.10	0.78–1.54	1.34	0.96–1.87	**1.57**	**1.30–1.91**	**2.16**	**1.18–3.94**	**1.53**	**1.41–1.67**
Influvac trivalent (Abbott) n = 4,597	NA	NA	NA	NA	**1.44**	**1.13–1.83**	1.25	0.75–2.07	**1.40**	**1.26–1.56**
Unknown/missing/other n = 95	1.64	0.55–4.87	1.76	0.26–11.72	NA	NA	NA	NA	0.52	0.47–0.59
2018/19	Incidence	95% CI	Incidence	95% CI	Incidence	95% CI	Incidence	95% CI	Standardised incidence	95% CI
Controls n = 21,999	5.48	4.20–7.02	5.10	4.20–6.15	2.64	2.40–2.94	1.01	0.80–1.27	2.45	2.30–2.62
Vaccinees n = 25,799	8.58	7.30–9.99	4.77	3.90–5.83	3.58	3.30–3.89	1.21	1.00–1.49	NA	NA
2018/19	IRR	95% CI	IRR	95% CI	IRR	95% CI	IRR	95% CI	Standardised IRR	95% CI
FluLaval quadrivalent (GSK) n = 4,637	**1.71**	**1.15–2.54**	0.92	0.55–1.53	**1.73**	**1.44–2.08**	1.35	0.50–3.66	1.03	0.93–1.15
FluMist quadravalent (AstraZeneca) n = 878	1.46	0.89–2.39	0.85	0.56–1.28	NA	NA	NA	NA	1.07	0.90–1.27
Fluviral trivalent (GSK) n = 11,625	**3.22**	**1.12–9.26**	1.03	0.39–2.73	1.12	0.93–1.35	1.02	0.72–1.43	**1.30**	**1.19–1.42**
Fluzone high dose trivalent (Sanofi Pasteur) n = 64	NA	NA	NA	NA	NA	NA	**5.70**	**1.86–17.49**	**1.50**	**1.41–1.67**
Fluzone quadrivalent (Sanofi Pasteur) n = 5,910	**1.50**	**1.10–2.06**	1.03	0.73–1.45	**1.51**	**1.25–1.84**	**2.54**	**1.36–4.76**	**1.56**	**1.44–1.70**
Influvac trivalent (Abbott) n = 2,437	NA	NA	NA	NA	0.94	0.67–1.32	1.40	0.79–2.47	1.02	0.92–1.14
Unknown/missing/other n = 248	2.43	0.65–9.05	NA	NA	1.81	0.95–3.47	NA	NA	**1.30**	**1.19–1.42**

CI: confidence interval; IRR: incidence rate ratio; NA: not applicable (in most cases, the vaccine in question did not cause any health events in the specific age group therefore an IRR could not be calculated. Fluzone high dose vaccine was only given to adults aged ≥ 65 years and Flumist was only given to children aged < 15 years).

^a^ A health event was defined by the respondent as of sufficient intensity to cause a medical consultation or work/school/day care absenteeism/prevent daily activities, or both.

The bold font indicates higher risk in vaccinees compared with controls.

### Age-standardised incidence rate ratios

Age-standardised incidence rate ratios for specific events by year are shown in Table 4. Systemic reactions (fever, fatigue, myalgia, malaise), nausea, vomiting, diarrhoea and stomach pain and headache/migraine were significantly more frequent among vaccinated participants.

**Table 4:** Incidence risk ratios for health events^a^ in vaccinees compared with controls, Canada 2017/18 and 2018/19 (n = 91,549)

Signs and symptoms	6 months–4 years IRR	95% CI	5–14 years IRR	95% CI	15–64 years IRR	95% CI	≥ 65 years IRR	95% CI	Total standardised IRR	95% CI
2017/18										
Fever (≥ 38 °C)	1.25	0.78–2.00	**2.11**	**1.17–3.79**	**1.45**	**1.02–2.06**	0.98	0.42–2.29	**1.47**	**1.24–1.74**
Stopped eating (children only^b^)	0.86	0.53–1.39	1.36	0.77–2.40	ND	ND	ND	ND	1.10	0.85–1.42
Sore throat	0.69	0.38–1.24	0.96	0.59–1.57	0.98	0.79–1.23	1.31	0.72–2.36	0.99	0.86–1.13
Feeling unwell/fatigue/ weakness/muscle aches/joint pain	0.66	0.41–1.04	1.09	0.70–1.67	**1.34**	**1.10–1.63**	1.37	0.82–2.28	**1.21**	**1.08–1.36**
Respiratory^c^	0.69	0.45–1.06	0.83	0.49–1.38	0.97	0.76–1.23	0.93	0.54–1.60	0.89	0.78–1.01
Gastrointestinal problems	1.33	0.72–2.45	1.22	0.72–2.06	1.31	0.97–1.77	**3.55**	**1.25–10.09**	1.41	0.78–1.01
Migraine/headache	0.93	0.35–2.49	0.92	0.57–1.49	**1.41**	**1.12–1.78**	1.59	0.83–3.04	**1.32**	**1.15–1.51**
2018/19										
Fever (≥ 38 °C)	**1.90**	**1.21–2.99**	**1.89**	**1.17–3.05**	1.22	0.88–1.69	1.73	0.80–3.75	**1.53**	**1.27–1.84**
Stopped eating (children only^b^)	0.03	0.00–0.20	NA	NA	NA	NA	NA	NA	0.01	0–0.08
Sore throat	1.39	0.74–2.59	0.55	0.36–0.86	0.88	0.72–1.07	1.64	0.99–2.72	0.90	0.79–1.03
Feeling unwell/fatigue/ weakness/muscle aches/joint pain	1.27	0.84–1.94	0.80	0.57–1.13	**1.36**	**1.15–1.60**	1.40	0.93–2.13	**1.26**	**1.13–1.40**
Respiratory^c^	1.33	0.88–2.01	0.59	0.39–0.90	1.01	0.82–1.24	1.33	0.82–2.14	0.99	0.87–1.13
Gastrointestinal problems	1.46	0.87–2.44	1.01	0.64–1.60	**1.60**	**1.19–2.15**	**2.24**	**1.03–4.90**	**1.46**	**1.23–1.74**
Migraine/headache	0.91	0.37–2.22	0.95	0.60–1.52	**1.24**	**1.02–1.51**	**2.24**	**1.23–4.11**	**1.25**	**1.09–1.43**

CI: confidence interval; IRR: incidence rate ratio; NA: not applicable; ND: not determined.

^a^ Health event was defined by the respondent as of sufficient intensity to cause a medical consultation or work/school/day care absenteeism/prevent daily activities, or both.

^b^ Children are aged 6 months–14 years .

^c ^Respiratory is defined as upper/lower tract infection symptoms, including asthma.

Allergic-like-illness, anaesthesia/paraesthesia, anaphylaxis, oculorespiratory syndrome, and seizures did not occur in both cases and controls.

The bold font indicates higher risk in vaccinees compared with controls.

### Emergency department visits and hospitalisations

Over both seasons 97/55,972 (0.2%) vaccinees and 50/35,577 (0.1%) controls reported an emergency department visit. While the majority of emergency department visits appeared to be conditions unrelated to vaccination (e.g. pneumonia, coccyx fracture) the following may have been vaccine related: cellulitis in vaccinated arm (n = 4 in vaccinees, 0 in controls), allergic-like reaction (n = 9 in vaccinees, one within the first hour following vaccination and six within the first 24 hours; 0 in controls). A total of nine vaccinees and nine controls required hospitalisation with a mean duration of 1.1 days for vaccinees and 3.4 days for controls. Four of the vaccinees and four controls were hospitalised for concurrent infections, two vaccinees and four controls for chronic medical conditions (e.g. diabetes, cancer, etc.), one vaccinee and one control for acute medical conditions (e.g. bowel obstruction, appendicitis) and one vaccinee for anaphylaxis following vaccination and one vaccinee for Henoch–Schonlein purpura.

### Onset and duration

Among vaccinees with a health event meeting the main outcome criteria, 37.6% (n = 682/1,814) had onset of their event within 24 hours of vaccination, whereas only 9.5% (n = 82/867) of controls reported onset of their event within the past 24 hours of receiving the control survey (p < 0.001). There was also a difference in event duration between controls and vaccinees. The event resolved within 72 hours for 54.6% (n = 991/1,814) of vaccinees vs 35.6% (n = 309/867) of controls (p < 0.001).

### Effect of health event on vaccine intentions

Among vaccinees who reported a health event on the 2018/19 survey (this question was not asked in 2017/18), 57% (486/857) stated they would be vaccinated next year, 16% were unsure (140/857) and 2.6% (22/857) would not be vaccinated again.

## Discussion

Among participants vaccinated with a seasonal influenza vaccine in 2017/18 and 2018/19 in Canada, a higher rate of health events causing absenteeism or preventing daily activities was noted in vaccinees vs controls while rates for medically-attended events were similar. This greater risk was associated with four seasonal influenza vaccines products in each year and two of the products were associated in both years. In both years, the difference in reported events was associated primarily with systemic symptoms (fever ≥ 38.0^◦^C, fatigue, myalgia), headache and gastrointestinal symptoms (nausea, vomiting). The onset and duration for the majority of these events were within 72 hours of vaccination and compatible with what we would expect for fever and other systemic reactions after seasonal influenza vaccines [13]. Reassuringly, reports of medically-attended events did not differ between years or between vaccinees and controls.

There are limited published data comparable to our results as most AEFI surveillance systems do not include a control group and/or collect only serious cases (i.e. hospitalisations or death) or do not collect information in all age groups. However, the AusVaxSafety system in Australia, while lacking a control group, does collect electronically self-reported data [11,14,15] on AEFI of any severity. This system sends a mobile text message from the healthcare provider 3 days after vaccination. Participants who report a medically-attended event then complete an online survey. Similar to our data, AusVaxSafety reported higher percentages of events among vaccinated children compared with adults [16], with, among all vaccinees (children and adults), systemic (fever) and gastrointestinal symptoms occurring most frequently [11]. Their 2015 and 2017 percentages for medically-attended events in children at 1.1% and 1.0%, were lower than ours (2.1% in both years), but covered a shorter duration of follow up (3 vs 7 days) [16,17]. Given the findings from AusVaxSafety and CANVAS, parents may benefit from improved counselling on expected symptoms following influenza vaccination and advice on potential treatments to minimise their effect.

CANVAS focuses on events occurring within the first 7 days following vaccination. While this time frame favours acute and local reactions over more complex immunologic processes, the system can send participants an additional survey, later during the year, should a more complex signal be detected through passive or active surveillance. Because CANVAS is based on self-report, we are unable to verify reported events and diagnoses with review of medical charts. Self-reporting of health events is subjective and may be subject to recall bias. However, self-report data have been shown to be a reliable proxy for healthcare utilisation and absenteeism for short recall periods of up to 1 month [18] and self-report of AEFI has been shown to describe more serious events than those reported by healthcare providers [19].

Our data have some limitations. We did have non-responders, especially in the control group, but our response rate of 67% for vaccinees and 49% for controls is higher than the average for online surveys (30%) and similar to that measured for in-person surveys (57%) [20]. We suspect the lower response rate in the control group represents those lost-to-follow up and reflects the time lag of almost 12 months between completion of the vaccine survey, when members of that group were recruited and the control survey. Non-responders were not contacted in 2017/18 or 2018/19. However, in past years of our surveillance, 10% of non-responders were randomly selected and contacted by phone with no differences found in event rates between those who responded online and those contacted by phone [9]. Selection bias may be a factor for our study. Individuals who seek vaccination at the earliest opportunity may differ in health seeking and reporting behaviour from those vaccinated later in the season. This is a necessary trade-off for surveillance designed to detect signals early in the vaccination campaign, but it should be noted. It is likely that a proportion of vaccinees have volunteered to participate in previous surveys, since the same vaccine providers are involved in recruitment each year. The effect that familiarity with the survey instrument might have on the accuracy or rate of reporting is unknown. Finally, sample size is an issue for identifying very rare adverse events (such as Guillain–Barré syndrome) or when comparing different vaccine products.

Although CANVAS was originally designed for use during influenza pandemics and has been used successfully in this setting [10], the value of annual repetition of the survey should be emphasised. Knowing the previous year’s crude rate of events on a weekly basis in both vaccinated and unvaccinated participants allows for immediate identification of a higher crude rate of AEFI reporting overall and by vaccine and thus can provide an early signal of an aberration in crude reporting rates. The surveillance is designed to detect signals; therefore, our main outcome was defined to include health events of sufficient severity to be clinically relevant and yet is broader than the serious adverse event (SAE) definition that used in other AEFI surveillance systems [21] to create a lower threshold for the captured events and the potential for signal detection. The event rates in the vaccinated group should be interpreted in the context of the control (unvaccinated) group. Indeed, this is the strength of this system as it provides a direct comparator for these events in a similar population. Another strength of the system is its ability to be rapidly scaled up and deployed to monitor new vaccines delivered in a mass immunisation setting. This would be applicable for vaccines used during outbreaks and pandemics. In fact, CANVAS was used to monitor the safety of a new meningococcal B vaccine during a mass immunisation campaign in Quebec [22]). The resource requirements (both personnel and financial) for a system such as CANVAS are minimal and likely within the annual surveillance budget for most countries with existing AEFI surveillance. Finally, engaging the public in influenza vaccine safety monitoring offers powerful knowledge translation of pharmacovigilance. Such engagement can change the norms around vaccine safety monitoring, ideally to the point where every individual who receives a vaccine will automatically respond yes or no about the occurrence of health events following receipt. Such transparent, participant-centred systems have the potential to increase vaccine confidence. CANVAS participants are able to check our study website throughout the data collection period for weekly updates and are emailed a one-page summary of results at the end of each season.

Most countries have passive AEFI surveillance. These generally inadequately sensitive systems may nevertheless generate safety signal especially for rare adverse events. However, in absence of a control group these systems cannot ascertain the association between these AEFI and the vaccine(s). The goal of the CANVAS network is to be complementary to existing systems routinely used to monitor the safety of influenza vaccines in Canada, while providing a greater sensitivity to capture safety signals and facilitate their assessment with its control group of unvaccinated respondents. Although a higher rate of systemic health events was reported among participants vaccinated with seasonal influenza vaccine in 2017/18 and 2018/19 seasons, these events were self-limited in nature; they were of the type and frequency to be expected with influenza vaccines.

## Supplementary Data

345000 Supplementary Figure 1
