# Supplementary material for: 2017/18 and 2018/19 seasonal influenza vaccine safety surveillance, Canadian National Vaccine Safety (CANVAS) Network
Source: Euro Surveill. 2020 Jun 4;25(22):1900470. doi: 10.2807/1560-7917.ES.2020.25.22.1900470 (PMC7336108; doi:10.2807/1560-7917.ES.2020.25.22.1900470)
Supplement: Supplementary Figure 1 [file 1900470_BETTINGER_Supplementary_Figure1.pdf]

Supplementary Figure 1: Flow of participants enrolled in the Canadian National Vaccine Safety network (CANVAS) 2017, 2018, Canada

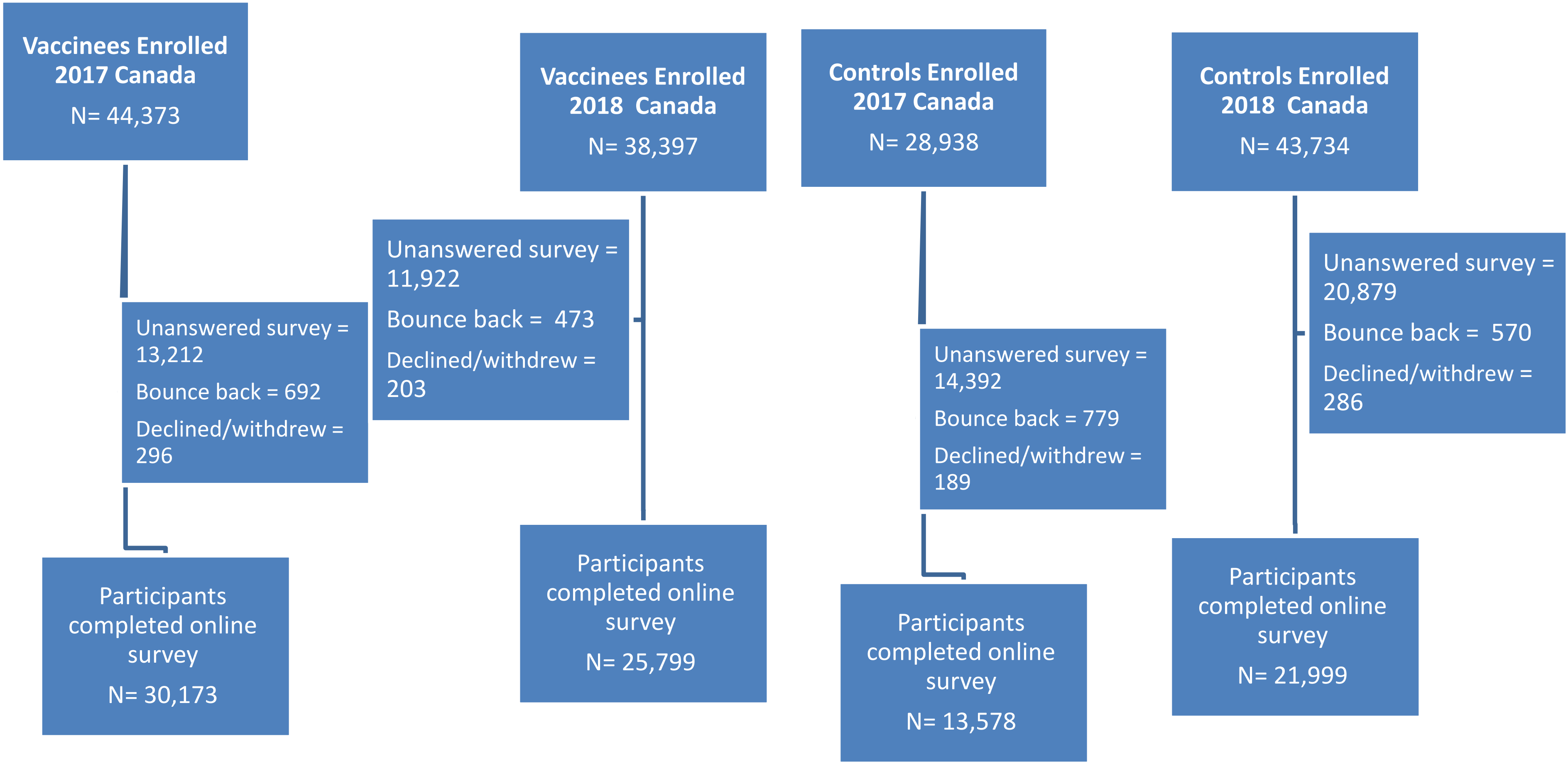

This supplementary material is hosted by *Eurosurveillance* as supporting information alongside the article 2017/18 and 2018/19 Seasonal Influenza Vaccine Safety Surveillance, Canadian National Vaccine Safety (CANVAS) Network, on behalf of the authors, who remain responsible for the accuracy and appropriateness of the content. The same standards for ethics, copyright, attributions and permissions as for the article apply. Supplements are not edited by *Eurosurveillance* and the journal is not responsible for the maintenance of any links or email addresses provided therein.
